# Wettability and Frictional Studies of PEEK Composites against Co-Cr Alloys with Surface Textures

**DOI:** 10.3390/polym15194006

**Published:** 2023-10-06

**Authors:** Xifang Zhang, Zhenqiang Yao, Haifeng Du, Jiacheng Song, Zhiyi Jin, Wei Xu

**Affiliations:** 1 College of Smart Energy, Shanghai Jiao Tong University, Shanghai 200240, China; xfzhang0103@sjtu.edu.cn (X.Z.); wei_xu@sjtu.edu.cn (W.X.); 2 School of Mechanical Engineering, Shanghai Jiao Tong University, Shanghai 200240, China; haifengdu@sjtu.edu.cn (H.D.); cheng.zi@sjtu.edu.cn (J.S.); jinzhiyi@sjtu.edu.cn (Z.J.); 3 State Key Laboratory of Mechanical System and Vibration, Shanghai 200240, China

**Keywords:** PEEK blends, Co-Cr alloy, laser textured surface, friction, wear, wettability

## Abstract

With the aim of promoting the qualities for total hip joint replacement, the wettability and tribological behaviors of PEEK composites pins with two sets of different fillers (PEEK/CF or PEEK/CF/PTFE/graphite) against Co-Cr alloy discs with five categories of surface textures (polished, orthogonal, spiral, r-θ, and orthogonal combined with spiral) were explored. It is revealed that the existence of CF in PEEK matrix increases the hydrophilicity in addition to the strength of PEEK, while the addition of PTFE increases the hydrophobicity of PEEK. The Co-Cr alloy discs with hydrophilic properties can be adjusted as hydrophobic, with the depth of textured grooves exceeding the critical sag height determined by the contact angle and the groove width. It can be concluded that PEEK/CF/PTFE/graphite composite has a lower wear rate than PEEK only reinforced with CF against Co-Cr alloy, both without surface texture and with shallow or deep grooves. The existence of shallow grooves on the disc surface could help the PEEK blends to achieve a steady friction against Co-Cr alloy in addition to collecting the worn debris. PEEK blend pins with 10 vol% CF, 10 vol% PTFE and 10 vol% graphite can achieve a lower friction coefficient of no more than 0.2 against Co-Cr alloy discs with shallow grooves around 3.5 μm in orthogonal or spiral textures.

## 1. Introduction

With the development of total hip joint replacement for artificial implants, the service life of prosthetic joints has exceeded 20 years; hence, an increasing number of young patients undergo joint orthopedic surgeries [1]. During total hip joint replacement, an artificial femur head made of CoCrMo alloys or ceramics is implanted into the hip joint together with a polymer insert liner made of ultrahigh molecular weight polyethylene (UHMWPE) or polyether ether ketone (PEEK) blends in acetabular bone [2]. The materials of the counterparts, the design parameters and the processing technologies play an important role in the friction behaviors and the wear performances of artificial hip joints [3].

In addition to the interface materials and their combinations, the design of artificial hip joint implants on structures and feature parameters as well as the surface morphologies of the counterparts have a crucial influence on prosthesis qualities. With the development of bionics and investigations of the tribological characteristics of artificial textured structures, surface textures could capture wear debris, decrease the actual contact area and form lubricating films to reduce friction and wear in different friction regimes [4,5,6]. The tribological results showed that a lotus leaf-like micro–nano hierarchical structure generated through chemical deposition on a copper substrate displays drag-reducing attributes [7]. Biomimetic shark skin textures were successfully fabricated by laser processing on a ZrO_2_/WS_2_ coating obtained through sol–gel technology, and dry friction reciprocating experiments showed that the average steady-state friction coefficient decreased by nearly 66% and the ball wear rate declined by approximately 82% compared with pure coatings [8]. Surface textures can be achieved through a variety of different processes and diverse manufacturing principles, such as cutting, abrasive machining, electrodischarge machining, electrochemical machining, ultrasonic-aided machining, lithography processing and laser beam machining [9,10,11].The hierarchical microdimples were conducted on a cylindrical surface using a dual-frequency surface texturing technology, and rotating cylinder-on-pin tribological tests exhibited that both the shape and variation in the length of the microdimple affect tribological performance under starved lubrication conditions [12]. Hara et al. [13] proposed an ultrasonic vibration-assisted turning (UVAT) technique to achieve periodic microtextures on various materials by adjusting the cutting and ultrasonic vibration parameters, and frictional tests demonstrated that the textured surface could enhance lubrication performance and reduce surface wear. Groove textures were performed on 40CrNiMoA via laser processing, and the ball-on-disc friction results showed that a larger width of the groove led to higher contact stress; thus, storing lubricant capacity and increasing contact stress should be balanced to obtain better tribological performance [14]. Textured surfaces with different densities, dimensions and depths were produced on chrome-bearing steels via laser processing, and pin-on-disc experiments showed that elliptical dimples with optimum geometrical parameters acted as a collector area for wear debris and wear reduction [15].

PEEK has been widely used as biomaterial due to its high mechanical strength, high melting temperature, excellent chemical resistance, self-lubrication, high wear resistance [16,17], good biocompatibility [18], and radiolucency [19]. Metal-on-polymer (CoCrMo-on-PEEK) tribological couples have been investigated for prosthesis applications owing to the mechanical properties of the metal and the abrasion resistance of the polymer counterpart. However, the submicron debris might induce the failure of joint replacement during the sliding contact interface [2]. Great research efforts have been made to improve metal-on-polymer tribological properties with PEEK blends. The introduction of graphite and polytetrafluoroethylene (PTFE) fillers significantly reduces the wear rates and coefficient of friction (COF) of PEEK composite coating in artificially implanted joints [1]. Experimental investigations have reported that the friction coefficient of PEEK reinforced with carbon fiber (CF) against Co-Cr-Mo alloy was effectively reduced and the wear rate was reduced compared with pure PEEK because CF could reduce shearing and bear the load preferentially [20,21]. Cao and Dong [22,23] found that a continuous and relatively uniform CF-PEEK transfer film on a textured WC–Co surface was produced via the microcutting effect of dimple texture edges on the material removal of the counterpart CF-PEEK pin, and the stable reduction in the friction coefficient was nearly 38.3% compared to the nontextured surface. Onodera and Haidar et al. [24,25] elucidated the mechanism of the excellent tribological performance of a PEEK polymer composed of PTFE by investigating the microstructure and function of the transfer film via XPS analysis with the argon etching technique, and the results showed that the transfer film to metallic surfaces was probably a gradient structure accompanied by a PTFE film on the topmost surface and a PEEK film mainly on the inside to reduce the friction. PEEK-MAX (Ti3SiC2, Ti3AlC2, and Cr2AlC) and PEEK-MoAlB composites were synthesized via hot pressing with ternary nanolaminates to explore the wettability, mechanical behavior, tribological behavior and antimicrobial behavior of PEEK matrix composites [26]. Experiments have demonstrated that PEEK composites with 0.25 wt% MoS_2_ added via ball milling and spark plasma sintering (SPS) had better lubricity and anti-wear performance in aqueous lubrication than PEEK/CF [27].

In this study, surface textures with different shapes and scales on L605 Co-Cr alloy discs were achieved using a femtosecond laser. PEEK blend pins were reinforced with 30 vol% CF or filled with 10 vol% CF, 10 vol% PTFE and 10 vol% graphite. The tribological behaviors of L605 discs against PEEK blend pins were investigated under dry sliding friction. With the aim of revealing the role of surface texture on the wear mode of a metallic femur head against artificial acetabular bone made of polymer and the film transfer mechanism between PEEK blends and L605 Co-Cr alloy, the tribological characteristics were assessed in terms of friction coefficients and wear rate.

## 2. Materials and Methods

### 2.1. Materials of PEEK Blend Pin and Co-Cr Alloy Disc

**Figure 1:**
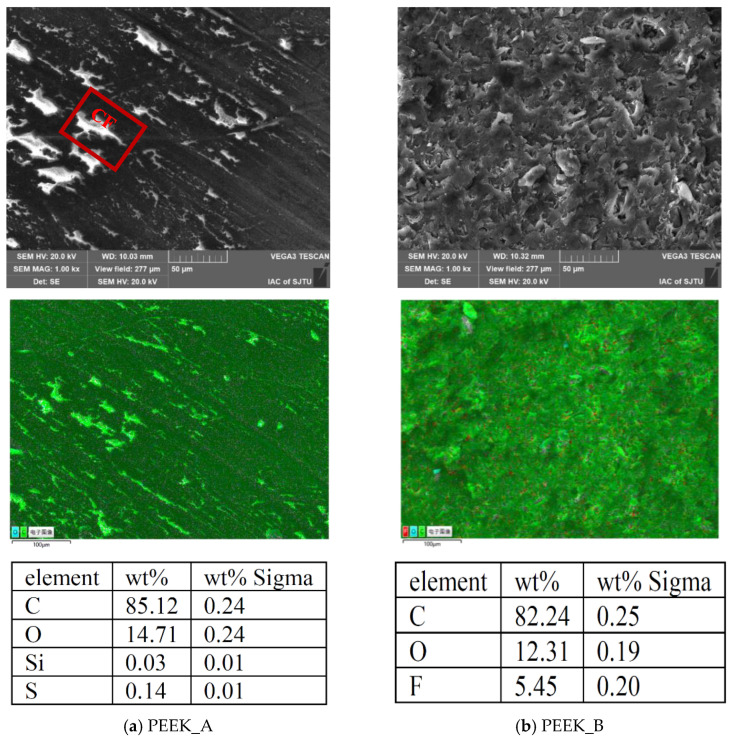
SEM images and EDS detection results of different PEEK blends: (**a**) PEEK_A and (**b**) PEEK_B.

Previous studies indicate that PEEK/CF composites exhibit the best anti-wear performance when the mass fraction of CF is in the range of 20–30% [28]. To examine the influences of fillers with mechanical and tribological advantages on the PEEK matrix, two kinds of PEEK blends (Ensinger Co., Ltd., Nufringen, Germany) with a diameter of 6 mm were employed as the pin, and the physical and mechanical properties of the PEEK blends are listed in Table 1. PEEK_A was reinforced with 30 vol% CF into the PEEK matrix, which was utilized to enhance the hardness, rigidity and dimensional stability of the composite material, while PEEK_B was composed of 10 vol% CF, 10 vol% PTFE, and 10 vol% graphite to improve the wear resistance with self-lubrication. The end face of each PEEK blend pin was precisely turned by INDEX G200 (Index Co., Ltd., Esslingen, Germany) with a surface roughness of approximately 0.40 μm. The SEM images and EDS inspection results for different PEEK blends are exhibited in Figure 1, in which the CF fillers were dispersed in the PEEK matrix like stripes, compared with the PTFE and graphite dispersed as flakes. The mass fractions of carbon and oxygen are higher in PEEK_A than in PEEK_B as there are more CF additives in PEEK_A.

L605 Co-Cr alloy (Leihua Alloy Co., Ltd., Shanghai, China) with a diameter of 40 mm was utilized as the disc with physical and mechanical properties shown in Table 2, which is mainly used in the manufacture of gas turbine engine components, high-temperature ball bearings, heart valves and stents in the medical industry owing to its high temperature resistance, corrosion resistance, abrasion resistance, and good biocompatibility. Before laser surface texturing, the L605 substrates were processed using waterproof abrasive papers and then polished using a polishing machine (MECATECH 250 SPI, PRESI, Eybens, France) to obtain a surface roughness *R*a of approximately 0.04 μm. All disc samples were thoroughly dried after ultrasonic cleaning for 30 min in acetone and alcohol.

### 2.2. Femtosecond Laser Processing on Disc Surfaces with Single and Multiple Patterns

To investigate the contact and frictional behaviors of PEEK blends against Co-Cr alloys with various surface textures, different patterns were prepared on alloy surfaces with femtosecond laser processing, while the PEEK composites retained smooth profiles due to their lower hardness.

**Figure 2:**
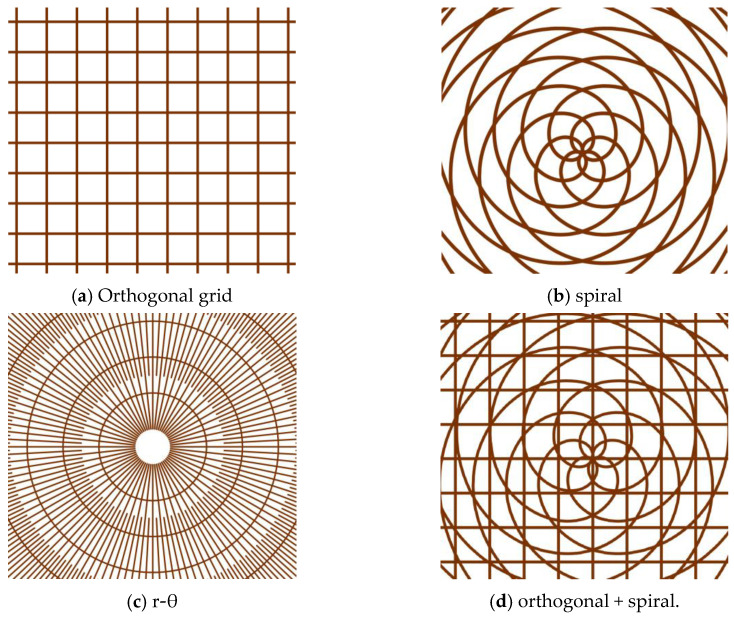
Schematic diagram of different surface textures scanned with femtosecond laser: (**a**) orthogonal grid, (**b**) spiral line, (**c**) r-θ and (**d**) orthogonal + spiral.

As shown in Figure 2, the orthogonal grid, spiral line, r-θ radioactivity pattern and combined textures of orthogonal grid overlaid with spiral line are designed to investigate the influence of texture shape and scale on tribological properties under dry friction with textured area ratios of 25%, 23%, 56% and 57%, respectively. A femtosecond laser power supply (Carbide-40 W, Light Conversion, Vilnius, Lithuania) with a maximum pulsed laser power of 40 W, base pulse frequency of 1 MHz, minimal laser spot diameter of 12 μm, laser wavelength of 1030 nm, pulse duration of 218 fs and pulse scanning speed of 1000 mm/s was selected to fabricate surface textures. The femtosecond laser processing parameters required to achieve single and combined textures on the L605 disc surface are listed in Table 3. At the same pulsed laser power of 20 W, single orthogonal and spiral textures were produced with laser scanning for 10 cycles, and r-θ textures were processed via scanning 20 times. Furthermore, the combined textures (orthogonally overlaid with spiral) were obtained with the combination of 20 W laser power with 100 scans and 5 W laser power with 10 scans. The laser processing path and parameters were scheduled and implemented through the computer control program. The profiles and topographies of surface textures were examined using a three-dimensional (3D) profilometer (VK-X3000, Keyence, Osaka, Japan) and a scanning electron microscope (SEM, VEGA3TESCAN, Brno, Czech Republic). The surface roughness was measured with the cut-off values of *λ*_s_ and *λ*_c_ being 2.5 μm and 0.8 mm, respectively.

### 2.3. Dry Friction Experiments

**Figure 3:**
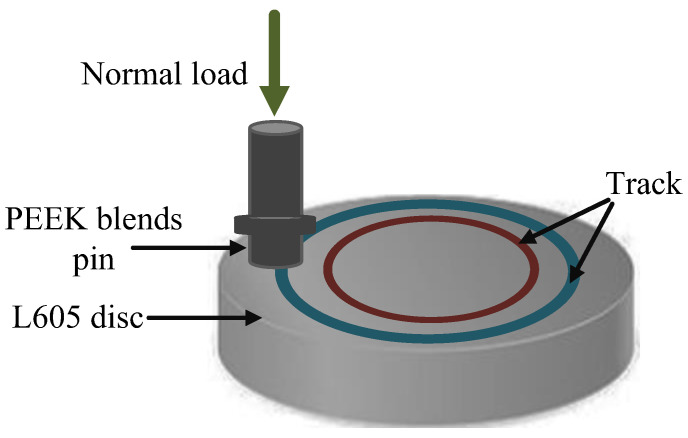
Schematic diagram of the pin-on-disc dry friction test.

The dry friction tests were carried out at room temperature using a tribometer (MFT-5000, RTEC, San Jose, Silicon Valley, California, USA) to analyze the frictional behaviors of textured and smooth disc surfaces against PEEK blend pins, where a normal load was applied to the pin for rotation along the disc, as illustrated in Figure 3. The friction test parameters of the pin-on-disc are listed in Table 4 with a rotational speed of 200 rpm and dry friction test time of 1800 s for all test settings. The specific pressure, *p*, was adjusted from 1.768 MPa to 2.476 MPa by varying the applied normal load from 50 N to 70 N, and the sliding velocity, *v*, was regulated from 0.209 m/s to 0.314 m/s along with the test track diameter changing from 20 mm to 30 mm. Therefore, the total *pv* value was controlled from 0.370 MPa·m/s to 0.777 MPa·m/s. Three repeated frictional experiments were prepared for each set of trials to calculate the average friction coefficients.

### 2.4. Wear Characteristics of PEEK Blends

The wear resistance of the PEEK composites not only reflects the toughness of the developed composites but also helps to determine their usage in biomedical applications. The specific wear rate, *w_s_*, referring to the worn volume over frictional work, is utilized to evaluate the wear properties of PEEK blends via the following equation [28,30]:

(1) $$w_{s}=\frac{\Delta V}{W_{f}}=\frac{\Delta m}{\rho F_{N}l}$$

where Δ*V* represents the worn volume, *W_f_* is proportional to the frictional work, *ρ* is the density of PEEK blends, *F_N_* denotes the applied normal load, *l* is the sliding distance and Δ*m* means the mass reduction, which was obtained by weighing the PEEK blends on a balance.

## 3. Results and Discussion

### 3.1. Topographic Characteristics of Laser-Textured Surfaces on Discs

**Figure 4:**
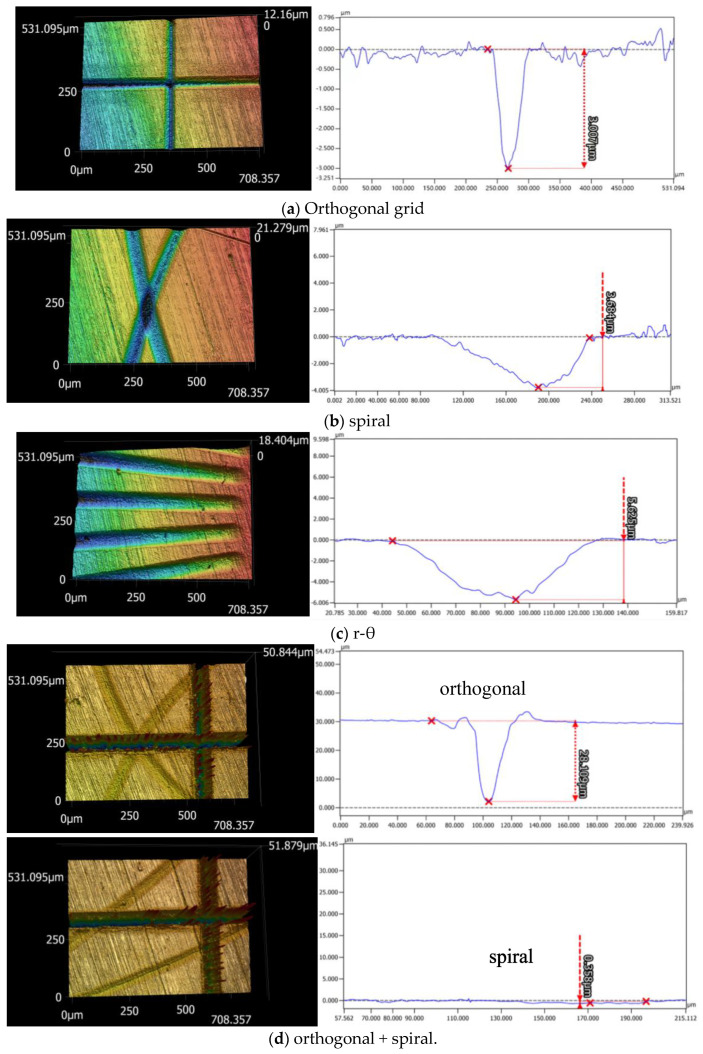
Three-dimensional topographies and 2D cross-section profiles of the single and multiscale textures: (**a**) orthogonal grid, (**b**) spiral line, (**c**) r-θ and (**d**) orthogonal + spiral.

**Figure 5:**
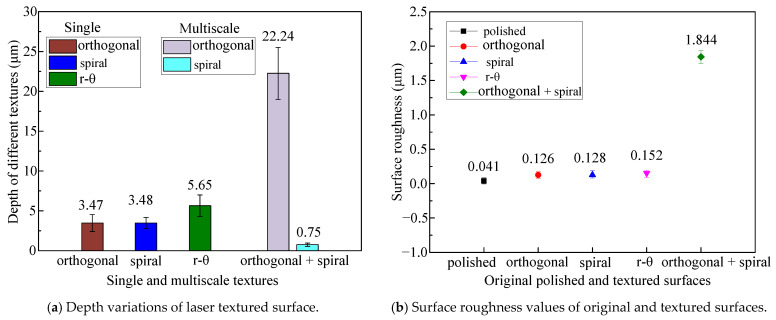
The depth variations and surface roughness values of the original polished and laser textured surfaces on the L605 disc: (**a**) depth variations of the laser textured surfaces and (**b**) surface roughness values of the original polished and textured surfaces.

**Figure 6:**
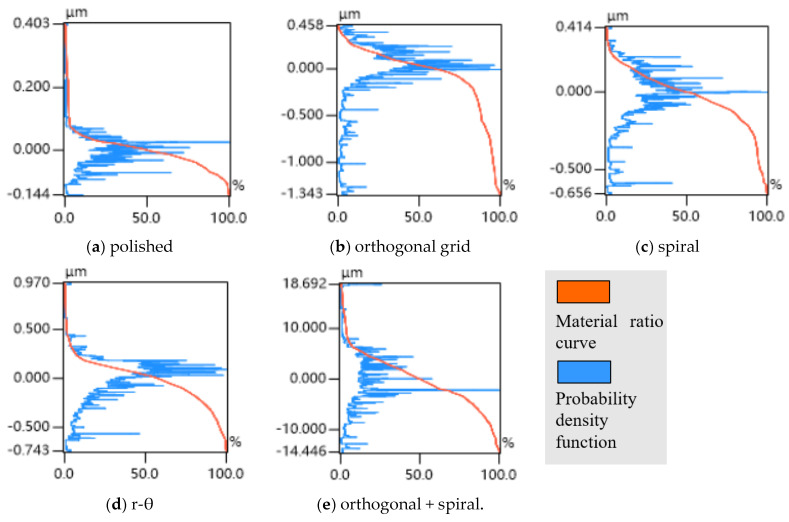
Material ratio curves (profile height–real material ratio) of the original polished and textured surfaces: (**a**) polished, (**b**) orthogonal grid, (**c**) spiral line, (**d**) r-θ and (**e**) orthogonal + spiral.

The pattern and groove profiles of the laser processing textures on Co-Cr alloy discs are characterized by the material ratio, groove depth, and surface roughness. The 3D dimensional topographies and 2D cross-section profiles of different shapes and scales for textures obtained via femtosecond laser processing on the L605 disc are shown in Figure 4. The depth variations of single and multiple textures generated with different combinations of laser machining parameters are exhibited in Figure 5a, where single orthogonal and spiral textures with a similar depth of 3.5 μm were achieved, r-θ textures with a depth of 5.65 μm were processed, and multiscale textures with a deep orthogonal depth of 22.24 μm overlaid with a shallow spiral depth of 0.75 μm were obtained. The average values and variations in surface roughness (*R*a) of single textured surfaces range between 0.126 and 0.152 μm, whereas the surface roughness value of the multiple textured surface reaches to 1.844 μm, as shown in Figure 5b. In Figure 6, the vertical axis represents the profile height, the blue curve represents the distribution ratio of the profile height, and the red curve along the horizonal axis denotes the real material ratio at different profile heights. The surfaces with different morphologies display different material ratio curves, indicating the ratio of the real contacting area over the nominal contacting area from peak to valley along the surface roughness depth. Figure 6 shows that the peak–valley value varies from 0.547 μm to 33.138 μm with the combined texture having the largest peak–valley value and root-mean-square (RMS) value.

**Figure 7:**
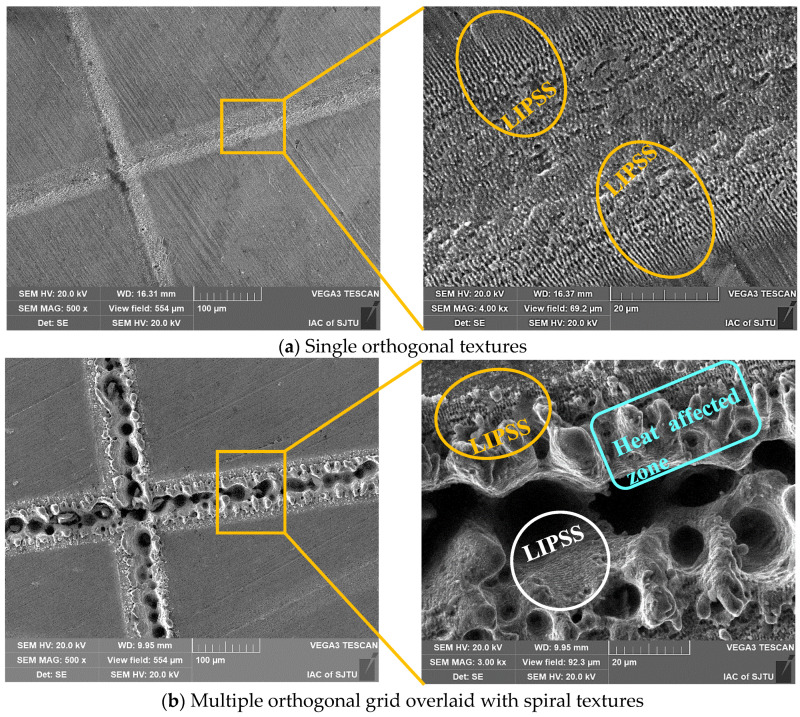
SEM images of single and multiple textures with LIPSS: (**a**) single orthogonal textures and (**b**) multiple orthogonal overlaid with spiral textures.

The SEM micrographs of single and multiple textured surfaces are displayed in Figure 7. It can clearly be seen that laser-induced periodic surface structures (LIPSS) appeared at the edge of the groove textures, which may be due to the Gaussian energy distribution, leading to lower laser energy accumulating at the margin of the groove textures. However, bulges can be observed on the edge of the deep orthogonal textures, resulting in poor surface roughness. This is attributed to more heat deposition at the edge of the deep grooves with layer-by-layer scanning 100 times, as well as the accumulation of molten metal ejected from the ablation pit during laser processing. The bottoms of grooves with different shapes and scales are not smooth but are distributed with microholes, which is due to the chaotic ablation process resulting from light reflection and refraction and structural self-assembly.

### 3.2. Wettability of PEEK Blend Pins and Co-Cr Alloy Discs

**Figure 8:**
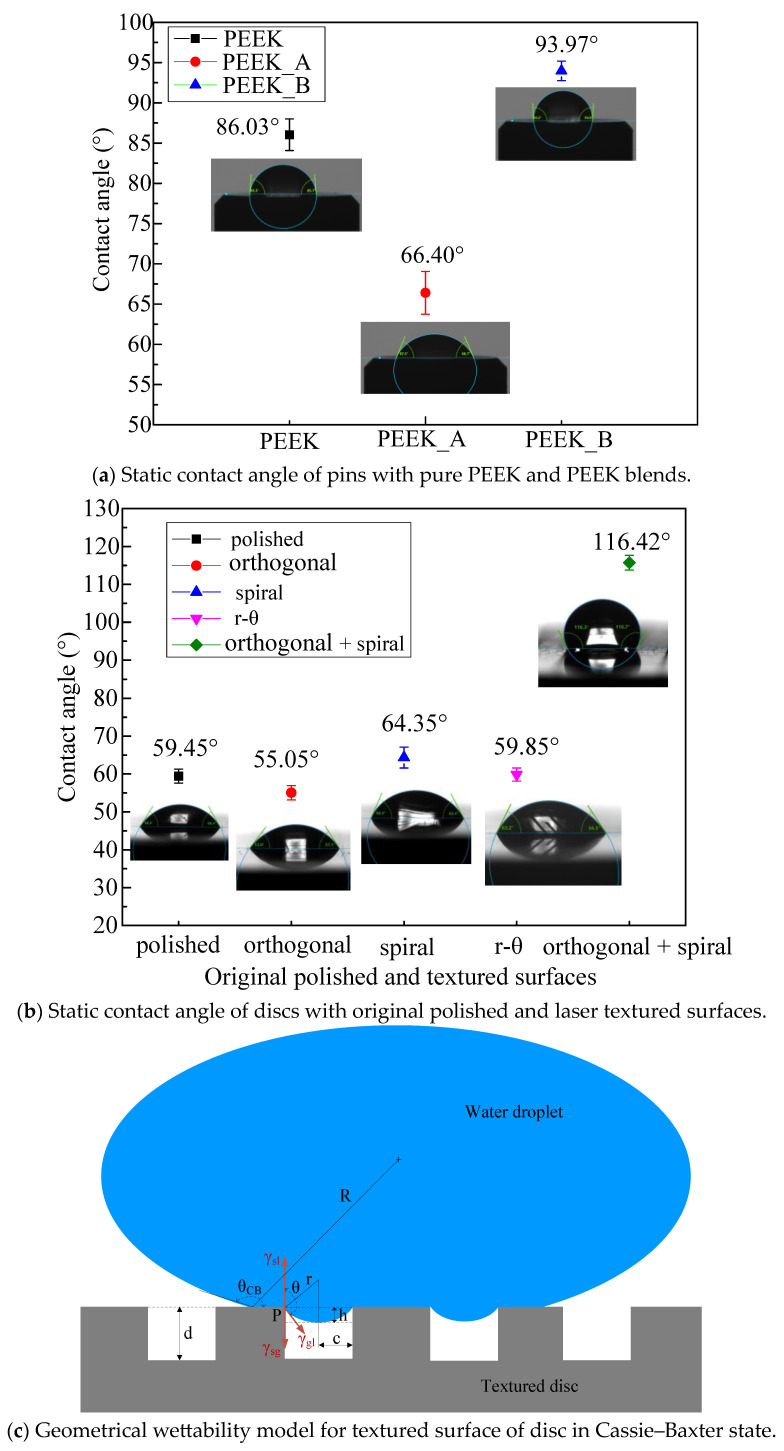
Static contact angle of PEEK pins and Co-Cr alloy discs as well as wettability model: (**a**) static contact angle of PEEK pins, (**b**) static contact angle of Co-Cr alloy discs and (**c**) wettability model for textured surface in Cassie–Baxter state.

Since the geometry of artificial hip joints and the bearing duty cycle (load and kinematics) of the human gait cycle do not always encourage hydrodynamic lubricated contact [31], the wettability of artificial joint surfaces with different textures should be taken into consideration. The contact angle (CA) was measured utilizing the tangent method at the endpoint of the droplet with a drop contact angle meter (DSA100, KRUSS, Hamburg, Germany) to explore the wettability of the PEEK blend pins and the textured surfaces of Co-Cr alloy discs. Deionized water droplets of 0.5 μL were deposited at 10 locations on each surface, and the static contact angles were calculated, as shown in Figure 8.

It can be seen from Figure 8a that pure PEEK had a CA of approximately 86.03°. The fillers of 30 vol% CF in the PEEK matrix (PEEK_A) decreased the CA to about 66.40°. The modification of hydrophilicity is due to the static CA of approximately 65.8° of CF in deionized water [32]. For PEEK_B, the addition of 10 vol% CF, 10 vol% PTFE and 10 vol% graphite in the PEEK matrix increased the CA to about 93.97°, which can be mainly attributed to the hydrophobic property of PTFE with a contact angle around 116° [33] whereas the CA of graphite is about 83.0° [34] close to that of pure PEEK. The addition of different fillers into the PEEK matrix can adjust the wettability of PEEK blends.

For the Co-Cr alloy discs, as shown in Figure 8b, the original polished surface exhibits hydrophilicity with a CA of 59.45°, and there is no significant difference in hydrophilicity between the polished surfaces and the single textured surface, where the CAs of the orthogonal, spiral, and r-θ textures were 55.05°, 64.35° and 59.85°, respectively. However, the multiple-textured surface with deep orthogonal grooves overlaying shallow spiral grooves displays hydrophobicity with a CA of 116.42°. Compared with Figure 5a, the textured surfaces with hydrophilicity have a shallow groove depth of no more than 6 μm, while the hydrophobic textured surface has a relatively deep groove depth of more than 22 μm. This can be explained by the Cassie–Baxter state [35] model, as shown in Figure 8c, where *R* is the radius from the center of the water droplet to the boundary edge of the water and Co-Cr alloy disc, the spherical radius *r* is the sag of the water droplet above the groove, *d* represents the depth of the textured groove, and *h* denotes the sag in the height of the droplet in the grooves. The contact angle of the textured disc surface with water is *θ*_CB_, while the contact angle of the polished disc surface with water is *θ*. At point *P*, where the groove and the pillar meet the water droplet, the surface tension parameters *γ*_sl_, *γ*_sg_ and *γ*_gl_ agree with Young’s equation. Based on the geometric relationship, the sag of water droplet *h* can be determined by contact angle *θ* and groove width *c* as follows:

(2) $$h=c\left|\frac{1-\sin\theta }{-\cos\theta }\right|$$

When the groove depth *d* exceeds *h*, the Cassie–Baxter state can be achieved to remain hydrophobic. This accounts for the wettability diversity of Co-Cr alloy discs with different depths of surface textures. Figure 4 shows that *c* is 37.5 μm and *θ* is 59.45°; hence, the critical value of *h* should be 10.24 μm. To achieve a textured hydrophobic surface on a Co-Cr alloy disc with *θ*_CB_ over 90°, the groove depth must surpass 10.24 μm.

### 3.3. Effects of Textured Surfaces on Frictional Properties

**Figure 9:**
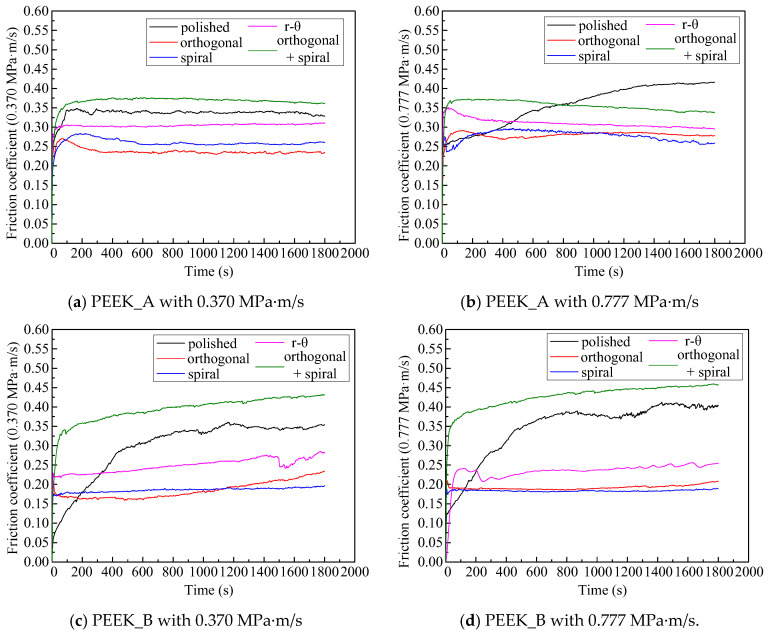
The evolution curves of the friction coefficient with sliding time for different PEEK pins and different *pv* values: (**a**) PEEK_A with 0.370 MPa·m/s, (**b**) PEEK_A with 0.777 MPa·m/s, (**c**) PEEK_B with 0.370 MPa·m/s and (**d**) PEEK_B with 0.777 MPa·m/s.

**Figure 10:**
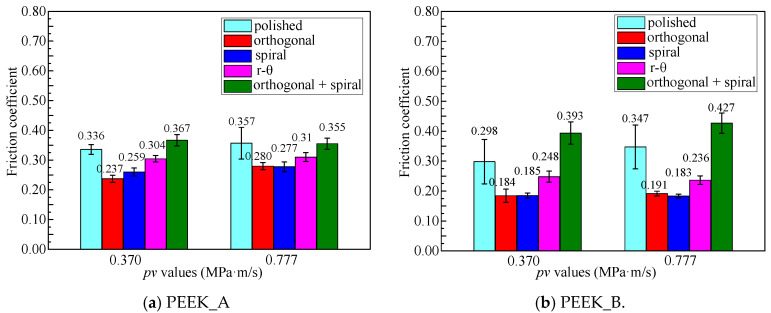
Average friction coefficient values of original polished and textured surfaces for different PEEK pins: (**a**) PEEK_A and (**b**) PEEK_B.

The frictional properties of PEEK blend pins against Co-Cr alloy discs are demonstrated in Figure 9 and Figure 10 in terms of two sets of *pv* values (0.370 MPa·m/s and 0.777 MPa·m/s) and five surface morphologies. It can be seen from Figure 9 that the friction coefficient of all textured disc surfaces initially increased and then basically reached a steady state with prolonged time, while the friction coefficient increased slowly for the original polished disc surface with sliding length, indicating a longer running-in period than that of textured discs. This can be attributed to the textured surfaces helping to reach a fast running-in procedure. It can be noticed from Figure 10 that discs against PEEK_B pins can achieve a lower friction coefficient than those against PEEK_A pins, apart from the disc with the combined texture. The friction coefficient from polished disc to spiral textured disc against PEEK_B brings about a relative reduction rate of 37.9% (0.370 MPa·m/s) and 47.3% (0.777 MPa·m/s).

This is attributed to the solid lubrication of PTFE and graphite in the PEEK matrix, as well as the hydrophobicity due to the deep groove effect on the disc with a combined texture. The discs with shallow single orthogonal or spiral textured surfaces helping to retain the hydrophilic property against PEEK_B blends with a solid lubrication effect can achieve the lowest friction coefficient of no more than 0.2.

It is well known from the binomial theorem of friction that the sliding friction force is composed of adhesion effects produced by molecular forces and ploughing effects generated by mechanical forces; therefore, the friction coefficient, *f*, can be expressed as follows [36]:

(3) $$f=\frac{\alpha A}{F_{N}}+\beta$$

where *A* is the real contact surface of the friction pairs, *F_N_* represents the applied normal load, and *α* and *β* are the molecular and mechanical force factors, respectively. The friction coefficient can also be characterized by the following formula [37]:

(4) $$f=f_{a}+f_{p}+f_{r}$$

where *f_a_* represents the adhesive effect related to the real contact area, *f_p_* denotes plastic deformation of ploughing and fracturing, and *f_r_* refers to the surface roughness associated with asperity interaction.

The polished surface could be considered a continuous surface leading to a large actual contact area, as well as an inability to capture wear debris. Consequently, the scraped particles embed into the contact surface between the PEEK blends against the L605 disc during dry sliding friction, leading to three-body wear [30] and a higher abrasive wear on the contact surface accompanied by a higher friction coefficient. This can be further validated by Figure 6a, in which the surface asperities are worn down at a high rate with the increase in material contacting area, indicating a high *f_p_* value.

The reasons for the multiple textured surfaces having a larger friction coefficient can be attributed on the one hand to the surface roughness sharply increasing due to the existence of protrusions associated with multiple laser ablations with as many as 100 scanning cycles with a higher *f_r_* value, and on the other hand to the difficulty in forming oxidation and adsorption films due to hydrophobicity (see Figure 8b), leading to aggravated adhesive wear effects with more CF debris and a higher *f_a_* value. In addition, Figure 6e shows a high worn down rate of the surface asperities as the material contacting area increase, indicating a high *f_p_* value.

The single orthogonal, spiral and r-θ textured surfaces have a positive effect on friction reduction due to their diverse groove structures, considered to be noncontinuous surfaces, which will reduce the real contact area and enhance the storage capacity of wear debris. In addition, the hydrophilicity of single textured surfaces more easily adsorbs water and oil in the air to form surface films, reducing the friction coefficient. It can be seen from Figure 6b–d that the single orthogonal and spiral textured surfaces have a rather low worn down rate of the surface asperities compared to r-θ textured surfaces. As the material contact area increases, they exhibit better influence on reducing the friction coefficient.

### 3.4. Wear Rate of PEEK Blends against Co-Cr Alloy Discs

**Figure 11:**
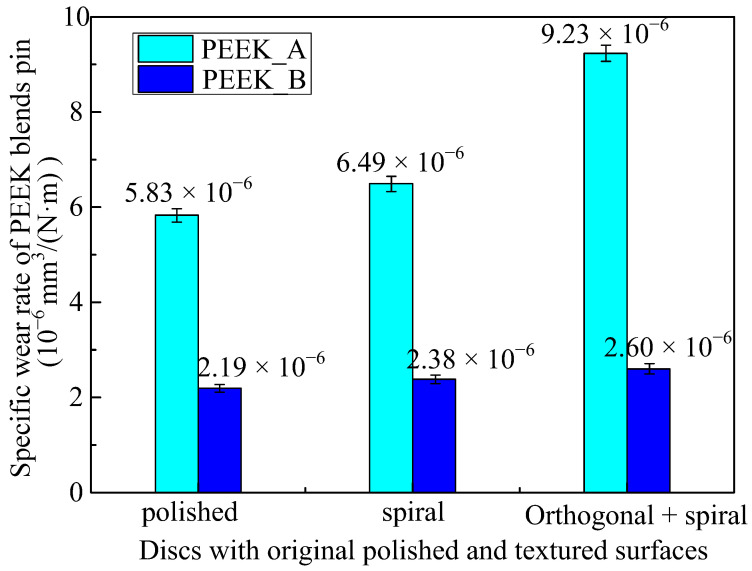
Specific wear rate of PEEK blend pins against discs with original polished and textured surfaces under the same *pv* value of 0.777 MPa·m/s.

Based on the frictional experiments shown in Figure 10, where discs with orthogonal, spiral, and r-θ surface textures exhibit nearly the same frictional behaviors against PEEK blend pins, the wear performances of two kinds of PEEK pins against Co-Cr alloy discs with three categories of surface textures (polished, spiral, orthogonal combined with spiral) under the same *pv* value of 0.777 MPa·m/s are depicted in Figure 11. It can be concluded that PEEK_B has a lower wear rate than PEEK_A, which verifies that the PEEK reinforced with PTFE and graphite fillers is beneficial for lubrication compared to the CF fillers. It can also be determined from Figure 11 that the wear rate has little difference, regardless of the PEEK_B pins against original polished or textured surfaces. The contributions of solid lubrication in the PEEK matrix are more important than the influence of surface textures on Co-Cr alloy discs. On the other hand, the PEEK_A pins against the textured surfaces have a higher wear rate than that of the original polished surface, especially the orthogonal overlaid with spiral textured surface, which has the highest wear rate. This is due to the existence of surface textures helping to enhance the formation of transferred film from PEEK blends to the discs, which is beneficial when it comes to achieving steady friction, as shown in Figure 9, but harmful to the wear of the PEEK blends.

### 3.5. Effects of Textural Morphology and Transferred Film

**Figure 12:**
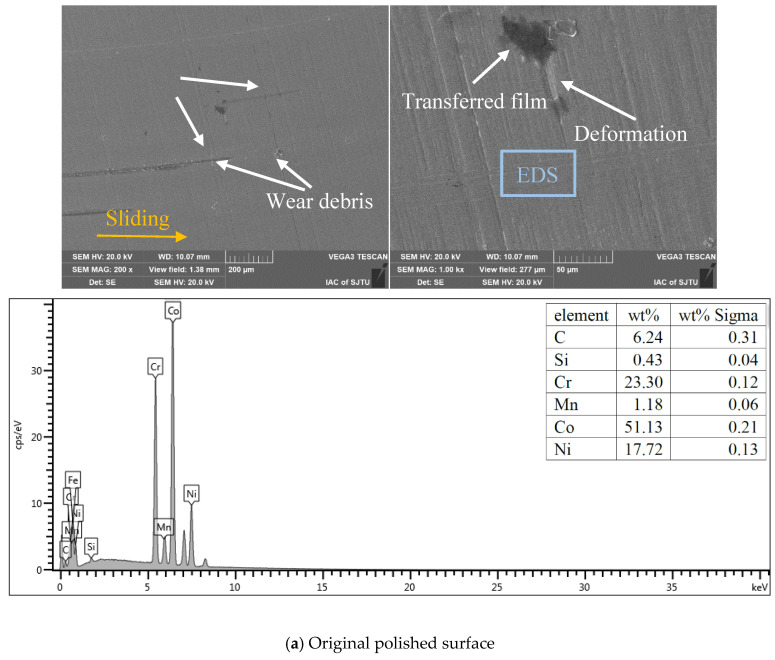

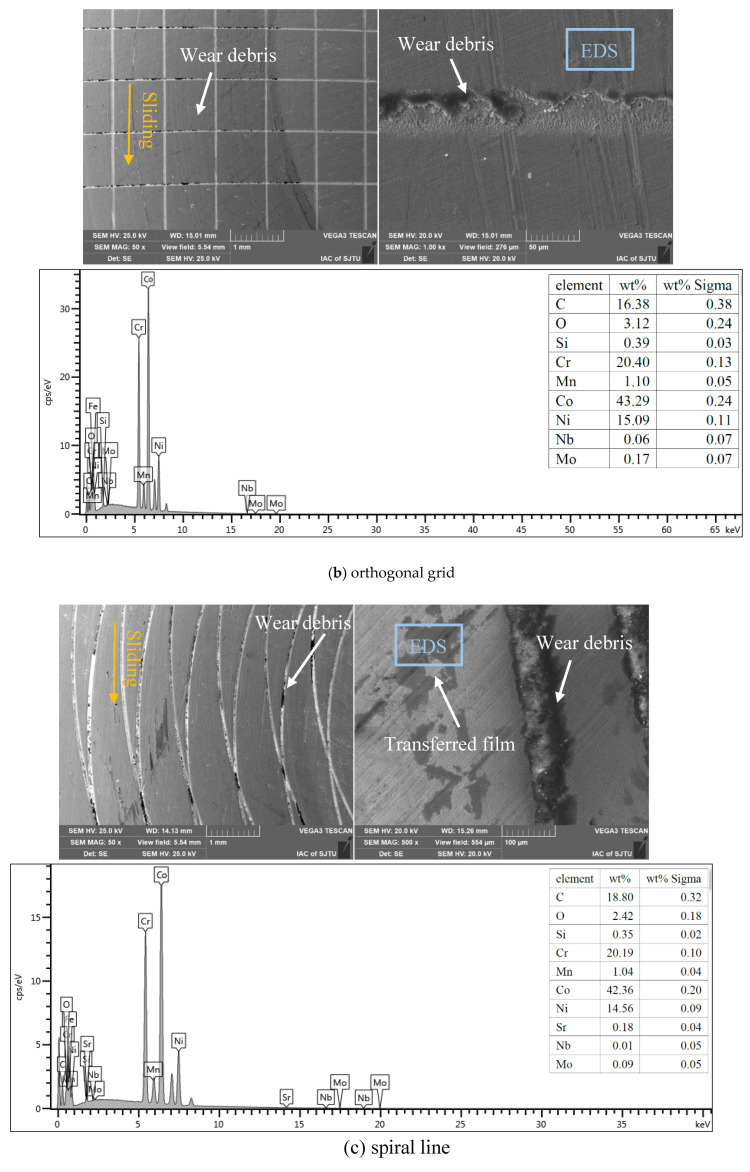

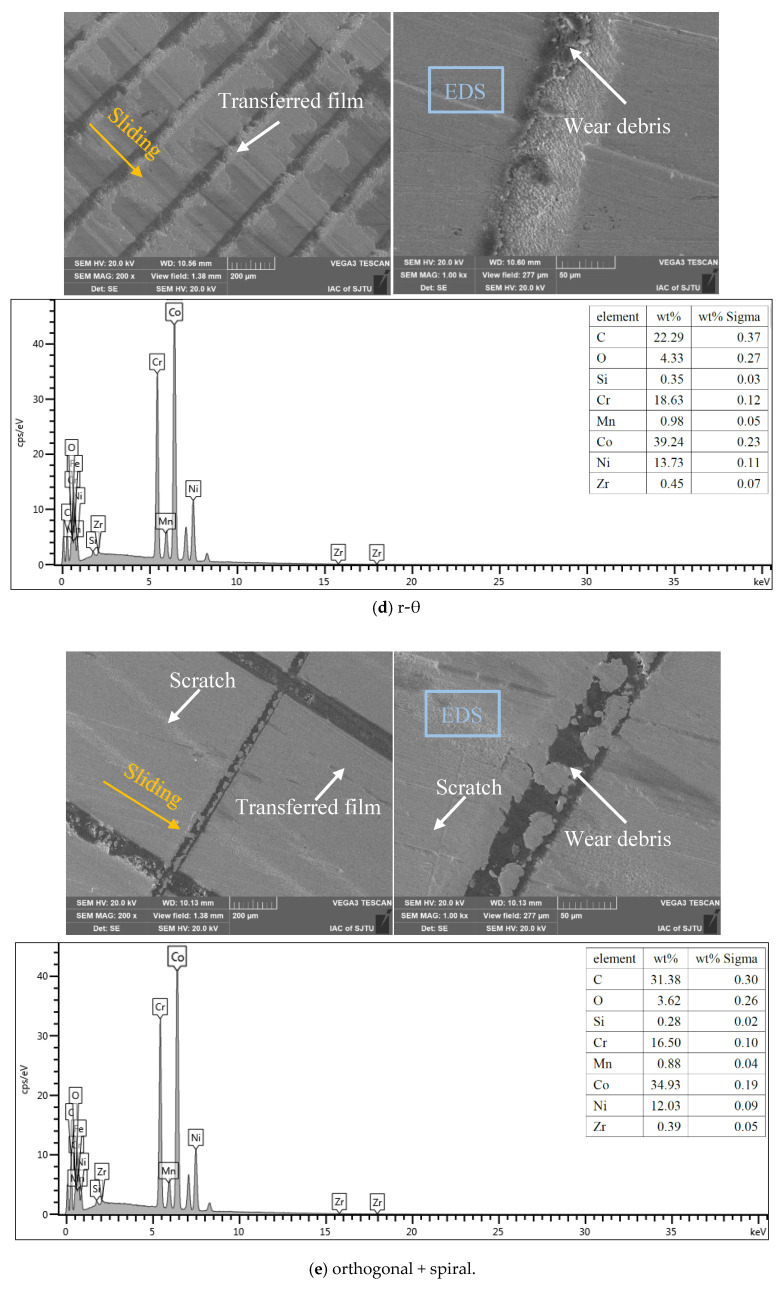
SEM images and EDS results of worn L605 polished and textured surfaces against PEEK_B: (**a**) polished, (**b**) orthogonal grid, (**c**) spiral line, (**d**) r-θ and (**e**) orthogonal + spiral.

As the evolution curves of the friction coefficient with prolonged time are similar for the L605 disc against the PEEK_A and PEEK_B pins, and PEEK_B has a better effect for friction reduction, consequently, the SEM morphologies and EDS for element content detection on the L605 Co-Cr alloy discs against the PEEK_B pin surfaces were examined to investigate the surface wear characteristics, as shown in Figure 12.

It can be seen from Figure 12a that the wear tracks of the polished nontextured surface have obvious scratches, wear debris accumulation and deformation, and there is very little carbon (6.24 w %) distributed on the L605 surface. Trapping wear debris during the friction process was very difficult for the nontextured surface, so the wear debris accumulation on the nontextured surface verified the three-body wear; that is, the hard L605 asperities were scraped off by the PEEK blends and embedded into the PEEK polymer, which can further cause severe scratches and microcutting on the smoothed surface. In turn, the edge bulges caused by scratches have the potential to scratch off the PEEK matrix material to the sliding interface, resulting in a thin and noncontinuous tribo-film. In addition, due to the real contact area of the smooth surface being larger than that of the textured surface, the friction force increased between the pin and polished disc surface and led to deformation on the nontextured surface. Therefore, the main wear mechanism of the smoothed surface may be severe abrasive wear followed by slight adhesive wear.

There are almost no scratches on the single orthogonal, spiral and r-θ textured surfaces, and the wear debris was trapped in the grooves, as shown in Figure 12b–d, which means that the good wear debris capacity of the different groove textures could obviously reduce the abrasive wear compared to the polished surface. Furthermore, the mass fractions of carbon and oxygen covered on the single orthogonal, spiral and r-θ are 19.5%, 21.22% and 26.62%, respectively, inferring that the PTFE, graphite and PEEK particles could be transferred to the disc surface to form a tribo-film near the grooves serving as solid lubricants, thereby aggravating the adhesive wear on the PEEK blend counterpart with more CF debris simultaneously. The great number of transferred films formed on the r-θ textured surface than on the single orthogonal and spiral textured surfaces also accounts for the higher friction coefficient of the r-θ textured surface than that on the single orthogonal and spiral textured surfaces.

Scratches and wear debris can be observed on the worn surface of the multiscale textured surface, as shown in Figure 12e, and the grooves are partly covered by wear debris. This may be because protrusions formed by laser heat deposition at the edge of the deep groove acted as a cutting tool to scratch more PEEK blend particles forming tribo-film, and the inference is also verified by the carbon and oxygen enrichment mass fraction of 35% covered on the surface. Furthermore, the protrusions experienced plastic fracture with reciprocating sliding, and then part of the fractured protrusions embedded into the PEEK blends, producing surface scratches, and part of the wear debris covered the grooves during sliding friction. Thus, the wear mechanism of the multiscale textured surface may be dominated by plastic deformation, adhesive wear and abrasive wear.

## 4. Conclusions

PEEK blends with different fillers are investigated in terms of their wettability and tribological performance against L605 Co-Cr alloy with laser processed surface textures. The conclusions are as follows:

(1) The wettability of PEEK blends can be tuned with different additives. Pure PEEK has a contact angle of approximately 86.03°, which can be decreased to approximately 66.40° by filling 30 vol% CF in the PEEK matrix and can be increased to approximately 93.97° with the addition of 10 vol% CF, 10 vol% PTFE and 10 vol% graphite in the PEEK matrix.

(2) The wettability of the L605 Co-Cr alloy can be adjusted with various surface textures. As the groove depth exceeds the critical sag height, which is determined by the groove width and contact angle, the Cassie–Baxter state can be achieved to maintain its hydrophobic state.

(3) PEEK blend pins with 10 vol% CF, 10 vol% PTFE and 10 vol% graphite can achieve a lower friction coefficient of no more than 0.2 against Co-Cr alloy discs with single orthogonal or spiral textures. The solid lubrication of PTFE and graphite as well as the hydrophilicity of the shallow groove depth account for the steady friction behavior.

(4) The addition of solid lubrication, including PTFE and graphite, into the PEEK matrix will decrease the adhesive wear. The combination of PEEK reinforced with PTFE and graphite and Co-Cr alloy discs with shallow surface textures will achieve better tribological characteristics.

## Figures and Tables

**Table 1:** Physical and mechanical properties of different PEEK composites claimed by the manufacturer.

Parameters	Standard [29]	PEEK_A	PEEK_B
Density (g/cm^3^)	ISO 1183	1.4	1.44
Melting point (°C)	ISO 11357	341	341
Thermal conductivity (W/mK) (23 °C)	ISO 22007-4	0.66	0.82
Tensile strength (MPa)	ISO 527	260	140
Bending strength (MPa)	ISO 178	380	190
Elastic modulus (MPa)	ISO 527	6000	5500
Ball indentation Hardness (MPa)	ISO 2039-1	298	250

**Table 2:** Physical and mechanical properties of the L605 Co-Cr alloy (ISO 5832-5 [30]).

Parameters	L605
Density (g/cm^3^)	9.10
Melting point (°C)	1330~1410
Thermal conductivity (W/mK) (100 °C)	10.5
Tensile strength (MPa)	1000
Bending strength (MPa)	500
Elastic modulus (GPa)	243
Brinell hardness (MPa)	282

**Table 3:** The processing parameters with the femtosecond laser for surface textures.

Laser Parameter Values	Surface Textures			
	Orthogonal	Spiral	r-θ	Orthogonal + Spiral
Wavelength (nm)	1030	1030	1030	1030
Pulse duration (fs)	218	218	218	218
Pulse frequency (kHz)	1000	1000	1000	1000
Laser spot diameter (μm)	12	12	12	12
Scanning speed (mm/s)	1000	1000	1000	1000
Pulsed laser power (W)	20	20	20	20 (grid), 5 (spiral)
Processing cycles	10	10	20	100 (grid), 10 (spiral)
Area density ratio (%)	25	23	56	57

**Table 4:** Dry friction test parameters between the PEEK blend pins and L605 disc.

Pin Material	PEEK_A	PEEK_A	PEEK_B	PEEK_B
Friction test number	1	2	3	4
Diameter of the pin, *d*_0_ (mm)	6	6	6	6
Diameter of the track, *d* (mm)	20	30	20	30
Rotational speed, *n* (rpm)	200	200	200	200
Friction test time, *t* (s)	1800	1800	1800	1800
Sliding velocity, *v* (m/s)	0.209	0.314	0.209	0.314
Sliding distance, *l* (m)	376.2	565.2	376.2	565.2
Applied normal load, *F*_N_ (N)	50	70	50	70
Specific press, *p* (MPa)	1.768	2.476	1.768	2.476
*pv* value (MPa·m/s)	0.370	0.777	0.370	0.777

## Data Availability

The datasets generated or analyzed during this study are available from the corresponding author upon reasonable request.

## References

[1] Liao Y., Cao L., Wang Q., Li S., Lin Z., Li W., Zhang P., Yu C. (2021). Enhanced tribological properties of PEEK-based composite coatings reinforced by PTFE and graphite. J. Appl. Polym. Sci..

[2] Díaz C., Fuentes G. (2017). Tribological studies comparison between UHMWPE and PEEK for prosthesis application. Surf. Coat. Technol..

[3] Özmen Y. (2016). Si3N4 as a biomaterial and its tribo-characterization under water lubrication. Lubr. Sci..

[4] Li X., Guo Z., Huang Q., Yuan C. (2022). Application of Bionic Tribology in Water-Lubricated Bearing: A Review. J. Bionic Eng..

[5] Sharma S.K., Grewal H.S. (2023). Tribological Behavior of Bioinspired Surfaces. Biomimetics.

[6] Hariharan G., Krishnamachary P.C., Binoj J.S., Mansingh B.B. (2023). Influence of SiC Nanoparticles Reinforcement in Areca/Tamarind Hybrid Biopolymer Composites: Thermo-mechanical, Tribological and Morphological Features. J. Bionic Eng..

[7] Lu Y. (2017). Fabrication of a lotus leaf-like hierarchical structure to induce an air lubricant for drag reduction. Surf. Coat. Technol..

[8] Li X., Deng J., Lu Y., Zhang L., Sun J., Wu F. (2019). Tribological behavior of ZrO2/WS2 coating surfaces with biomimetic shark-skin structure. Ceram. Int..

[9] Brinksmeier E., Karpuschewski B., Yan J., Schönemann L. (2020). Manufacturing of multiscale structured surfaces. CIRP Ann..

[10] Vishnoi M., Kumar P., Murtaza Q. (2021). Surface texturing techniques to enhance tribological performance: A review. Surf. Interfaces.

[11] Rosenkranz A., Costa H.L., Baykara M.Z., Martini A. (2020). Synergetic effects of surface texturing and solid lubricants to tailor friction and wear—A review. Tribol. Int..

[12] Ali S., Kurniawan R., Chul P.G., Ko T.J. (2023). Tribological properties of hierarchical micro-dimples produced on a cylindrical surface by dual-frequency texturing. Friction.

[13] Hara K., Fukuda T., Taguchi K., Isobe H. (2022). Surface Texturing Technique Based on Ultrasonic Turning for Improving Tribological Properties. Int. J. Autom. Technol..

[14] Lin X., Li J., Xie S., Xia R., Liu J., Luo L. (2022). Effect of groove textures on tribological properties of 40CrNiMoA steel under starved grease lubrication. Surf. Topogra-Metro..

[15] Joshua S.P., Babu P.D. (2022). Friction and Wear Characteristics for Laser Surface Textured Rolling Bearing Steel: Effect of Pattern Density, Depth, and Area. Trans. Indian Inst. Met..

[16] King J.A., Tomasi J.M., Klimek-McDonald D.R., Miskioglu I., Odegard G.M., King T.R., Sutherland J.W. (2018). Effects of carbon fillers on the conductivity and tensile properties of polyetheretherketone composites. Polym. Compos..

[17] Dufils J., Faverjon F., Héau C., Donnet C., Benayoun S., Valette S. (2017). Combination of laser surface texturing and DLC coating on PEEK for enhanced tribological properties. Surf. Coatings Technol..

[18] Geng Y.-M., Ren D.-N., Li S.-Y., Li Z.-Y., Shen X.-Q., Yuan Y.-Y. (2020). Hydroxyapatite-incorporation improves bone formation on endosseous PEEK implant in canine tibia. J. Appl. Biomater. Func..

[19] Kurtz S.M., Devine J.N. (2007). PEEK biomaterials in trauma, orthopedic, and spinal implants. Biomaterials.

[20] Zhao X., Xiong D., Wu X. (2017). Effects of Surface Oxidation Treatment of Carbon Fibers on Biotribological Properties of CF/PEEK Materials. J. Bionic Eng..

[21] Pan L., Yapici U. (2016). A comparative study on mechanical properties of carbon fiber/PEEK composites. Adv. Compos. Mater..

[22] Cao H., Dong X., Qu D., Dong C., Zhao C., Sun D., Gu L., Wu B. (2022). Transfer film growth of continuous carbon fiber reinforced thermoplastic poly(ether ether ketone) facilitated by surface texture during dry sliding. J. Mater. Sci..

[23] Dong X., Dong C., Wu B. (2022). Insight Into Surface Texture-Induced Dual Effects on Friction of WC-Co Dry Sliding Against Continuous Carbon Fiber-Reinforced Thermoplastic and Thermosetting Composites. Tribol. Trans..

[24] Onodera T., Nunoshige J., Kawasaki K., Adachi K., Kurihara K., Kubo M. (2017). Structure and Function of Transfer Film Formed from PTFE/PEEK Polymer Blend. J. Phys. Chem. C.

[25] DHaidar D.R., Alam K.I., Burris D.L. (2018). Tribological Insensitivity of an Ultralow-Wear Poly(etheretherketone)–Polytetrafluoroethylene Polymer Blend to Changes in Environmental Moisture. J. Phys. Chem. C.

[26] Javaid S., Dey M., Kaabouch N., Gupta S. (2020). On the potential of polyetheretherketone matrix composites reinforced with ternary nanolaminates for tribological and biomedical applications. J. Appl. Polym. Sci..

[27] Hou X., Bai P., Li J., Li Y., Cao H., Wen X., Meng Y., Ma L., Tian Y. (2023). MoS2 reinforced PEEK composite for improved aqueous boundary lubrication. Friction.

[28] Friedrich K., Lu Z., Häger A. (1993). Overview on polymer composites for friction and wear application. Theor. Appl. Fract. Mech..

[29] Jin Z., Yao Z., Sun Y., Shen H. (2022). Loading capacity of PEEK blends in terms of wear rate and temperature. Wear.

[30] Liu H., Leng Y., Wan G., Huang N. (2011). Corrosion susceptibility investigation of Ti–O film modified cobalt-chromium alloy (L-605) vascular stents by cyclic potentiodynamic polarization measurement. Surf. Coat. Technol..

[31] Boedo S., Booker J.F. (2013). A Novel Elastic Squeeze Film Total Hip Replacement. J. Tribol..

[32] Qiu S., Fuentes C.A., Zhang D., Van Vuure A.W., Seveno D. (2016). Wettability of a Single Carbon Fiber. Langmuir.

[33] Xiong Z., Lai Q., Lu J., Qu F., Yu H., Chen X., Zhang G., Zhang W., Zhao S. (2023). Silanization enabled superhydrophobic PTFE membrane with antiwetting and antifouling properties for robust membrane distillation. J. Membr. Sci..

[34] Khalkhali M., Kazemi N., Zhang H., Liu Q. (2017). Wetting at the nanoscale: A molecular dynamics study. J. Chem. Phys..

[35] Cai Y., Chang W., Luo X., Qin Y. (2018). Superhydrophobicity of microstructured surfaces on zirconia by nanosecond pulsed laser. J. Micromanuf..

[36] Breki A., Kurakin M., Liashenko D., Moskvin S., Balakin S. (2020). Laws carbonite friction VK8 solid alloy in water environment. Mater. Today Proc..

[37] Zhan X., Yi P., Liu Y., Xiao P., Zhu X., Ma J. (2019). Effects of single- and multi-shape laser-textured surfaces on tribological properties under dry friction. Proc. Inst. Mech. Eng. Part C J. Mech. Eng. Sci..

